# Phages against Pathogenic Bacterial Biofilms and Biofilm-Based Infections: A Review

**DOI:** 10.3390/pharmaceutics14020427

**Published:** 2022-02-16

**Authors:** Siyu Liu, Hongyun Lu, Shengliang Zhang, Ying Shi, Qihe Chen

**Affiliations:** Department of Food Science and Nutrition, Zhejiang University, Hangzhou 310058, China; siyuliu@zju.edu.cn (S.L.); luhongyun@zju.edu.cn (H.L.); 12113064@zju.edu.cn (S.Z.)

**Keywords:** bacterial biofilm, phage, pathogenic bacterial biofilm control, phage therapy, biofilm-based infections

## Abstract

Bacterial biofilms formed by pathogens are known to be hundreds of times more resistant to antimicrobial agents than planktonic cells, making it extremely difficult to cure biofilm-based infections despite the use of antibiotics, which poses a serious threat to human health. Therefore, there is an urgent need to develop promising alternative antimicrobial therapies to reduce the burden of drug-resistant bacterial infections caused by biofilms. As natural enemies of bacteria, bacteriophages (phages) have the advantages of high specificity, safety and non-toxicity, and possess great potential in the defense and removal of pathogenic bacterial biofilms, which are considered to be alternatives to treat bacterial diseases. This work mainly reviews the composition, structure and formation process of bacterial biofilms, briefly discusses the interaction between phages and biofilms, and summarizes several strategies based on phages and their derivatives against biofilms and drug-resistant bacterial infections caused by biofilms, serving the purpose of developing novel, safe and effective treatment methods against biofilm-based infections and promoting the application of phages in maintaining human health.

## 1. Introduction

The increasing occurrence of antibiotic resistance in pathogenic bacteria has posed serious threats to the clinical, medical and food industries [1]. Bacterial biofilm formation is considered to be one of the resistance mechanisms against antibiotics, which increases the virulence to be more pathogenic [2]. According to the statistics, up to 80% of recurrent microbial and chronic infections in humans are related to the formation of bacterial biofilm [3]. The morphology and physiological functions of bacteria in biofilms are entirely different from those of planktonic bacteria free in suspension, allowing bacteria within biofilms to be upwards of 1000-fold more resistant to conventional antibiotic treatments and host immune responses as compared to the planktonic cells [4,5]. With the co-evolutionary adaptation of notorious human pathogens to hosts and the abuse of antibiotics in modern clinical medicine, intrinsic bacterial resistance to antibiotics has globally risen to a high-risk level [6]. Hence, efficient alternative therapeutic strategies are urgently needed to prevent pathogenic bacterial biofilm formation and control biofilm-based infections.

Phages are viruses found in almost every environment, which may persist as intracellular parasitic deoxyribonucleic acid (DNA) or require bacteria as a host for replication and cause bacterial lysis [7]. Research on phages as therapeutics began in the 1910s but was largely forgotten in the era of antibiotics during World War II [8]. There has been renewed interest in phage therapy in the past few years amid the emergence of antibiotic-resistant bacteria and a global supply shortage of newly developed antibiotics [9]. Recent studies have shown that phage therapy can be one of the most promising alternative treatment options for antibiotic-resistant pathogens, which is more effective than antibiotics against bacterial infections [10].

This paper mainly reviews the composition, architecture and formation process of bacterial biofilms, briefly introduces the classification and two major life cycles of phages, analyzes interaction mechanisms between phages and bacterial biofilms, and summarizes several phage-based applications for the control of bacterial biofilms and treatment of drug-resistant bacterial infections caused by biofilms, including phage cocktails, the use of phage-derived enzymes, and the combination of phages and/or their derivatives with antibiotics, nanoparticles as well as chemical disinfectants.

## 2. The Bacterial Biofilm

### 2.1. The Composition and Architecture of the Bacterial Biofilm

Bacterial biofilms refer to the extremely complex and highly structured communities encapsulated in self-produced extracellular polymeric substances (EPSs) that contain cells in distinct physiological and morphological states, which is irreversibly attached to biotic or abiotic surfaces [11,12]. In most bacterial biofilms, microorganisms account for only 10% of the dry weight, whereas the proportion of EPSs accounts for more than 90% [13]. An EPS, with a hydrated, gelatinous, three-dimensional architecture, consists of a variety of extracellular polysaccharides, proteins, lipids, nucleic acids (extracellular DNA and RNA), and other biomolecules, providing the mechanical stability of biofilms, protecting adhering bacteria against environmental attacks, and restricting the entry of antibiotics [14]. Though the exact composition and structure of bacterial biofilms vary greatly with bacterial species, nutrient availability and environmental conditions, extracellular polysaccharides and proteins are common underlying structural components of diverse bacterial biofilms [15]. Extracellular polysaccharides have been proven to possess strong metal binding and complexation potential, which can interact with divalent cations such as calcium and magnesium as well as zinc, promoting microbial adhesion to surfaces and the cohesion of biofilms, and providing a fundamental structural function for the integrity to the matrix [16,17]. Proteins have been identified as an indispensable major component of biofilms, undoubtedly having important functions in induced inflammation and biofilm maintenance [18]. In addition, proteins can also participate in the degradation of biofilms as extracellular enzymes, promoting the release of biofilm-resident bacteria and the formation of new biofilms [19]. Lipids make up a relatively low proportion in biofilms, but they are able to bind proteins to form lipoproteins, which play a crucial role in maintaining cellular integrity, establishing infections, and promoting biofilm formation [20]. In addition, the presence of lipids provides an important property for EPS, namely hydrophobicity. Extracellular DNA (eDNA) is a recently uncovered component almost ubiquitous in biofilms and has been implicated in the maturation of biofilms through interactions with other molecular components, such as exopolysaccharides, lipoproteins, and amyloidogenic peptides, helping to organize and stabilize the structures of biofilms [21,22]. Presumably, there are other cellular components presented in biofilms, and further research on their role in biofilm would be necessary.

### 2.2. The Formation of Bacterial Biofilm

**Figure 1:**
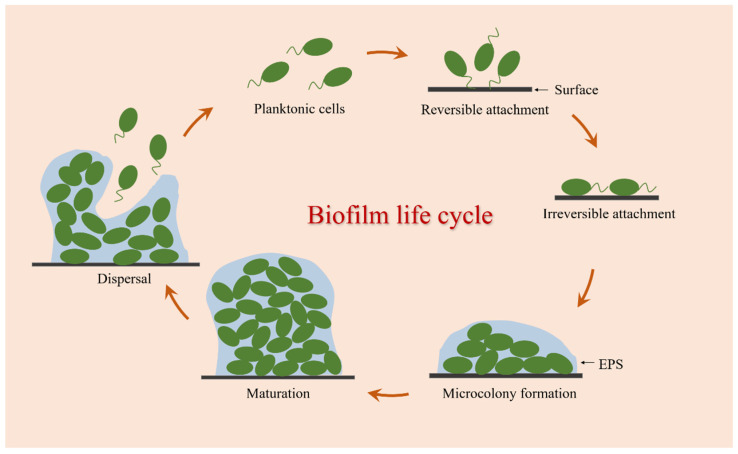
Biofilm formation process.

The formation of bacterial biofilm is a complex and dynamic process involving various physical, chemical, and biological processes. It is generally believed that the biofilm life cycle can be established mainly through the following stages: reversible attachment, irreversible attachment, microcolony formation, maturation, and dispersal (Figure 1).

Biofilm formation starts with a short and successive process of the adhesion detachment of bacterial cells, which is called reversible attachment [23]. At this stage, bacteria can sense and attach to biotic or abiotic surfaces by using a variety of extracellular organelles and proteins, and this interaction is primarily mediated by interfacial electrostatic forces and van der Waals forces, allowing bacteria to continue to form biofilms or return to the planktonic state from the contact surface [24]. The second process is the key turning point of bacteria cells from free state to biofilm called irreversible attachment. With an increasing number of adhesion bacteria, these attached cells begin to synthesize adhesin molecules, namely EPS, which can help facilitate adhesion between cells and surfaces [25]. Following irreversible attachment, bacterial cells begin to divide and grow into small aggregates of microorganisms called microcolonies [26]. In this stage, some genes related to biofilm formation are upregulated and expressed, and a large number of EPS are secreted for the adhesion, cohesion, and protection of microcolony clusters [27,28]. Then comes the maturation process. With the continuous multiplication of microcolonies and the increased secretion of EPS, these small cell clusters eventually develop and mature into the three-dimensional structure of biofilms. During this process, the colonies in the biofilm can transport substances such as water, nutrients, and metabolites, showing greater resistance to mechanical stresses and adverse environmental factors. Biofilm dispersal is the final stage of biofilm formation, which is an active process triggered by the deterioration of local conditions within biofilms [29]. This process is critical for the propagation and self-renewal of the community, allowing bacterial cells to actively escape from mature biofilms, and these released planktonic cells can diffuse into the bulk fluid to spawn novel biofilms in new locales [30]. Therefore, dispersion is not only the last stage of biofilm development but also the beginning of another biofilm life cycle.

## 3. Interactions between Phages and Bacterial Biofilms

### 3.1. Phages Infect Bacterial Biofilms

Phages are one of the most abundant and ubiquitous biological entities on our planet, which were discovered independently in the early twentieth century by Frederick Twort and Félix d’Hérelle [31]. They live everywhere and can be commonly found in places teeming with bacterial communities, such as wastewater, dirt, and the guts of animals. It is estimated that there are more than 10^31^ phage particles present in nature. Phages are highly specific and non-toxic, meaning that they exclusively infect bacteria and pose no threat to the cells of higher organisms [32]. Besides, when they act on the target pathogens, they do not damage the normal microflora of the host [33].

Phages are tiny and are composed of proteins and nucleic acids. Proteins are considered to be the coats of nucleic acids that determine the morphology of phages. Additionally, the genetic material of phages consists of double-stranded or single-stranded DNA or RNA [34]. Studies have shown that phages can be tailed, polyhedral, filamentous, or polymorphous. It has been reported that about 96% of phages are double-stranded DNA genomes with tailed morphology, belonging to the order of *Caudovirales* [35]. Based on the tail structure, the *Caudovirales* order can be categorized into three families: *Myoviridae* (phages with a contractile tail, such as T4-like phages), *Siphoviridae* (phages with a non-contractile long tail, such as T5-like phages), and *Podoviridae* (phages with a short tail, such as T7-like phages) [36,37,38].

**Figure 2:**
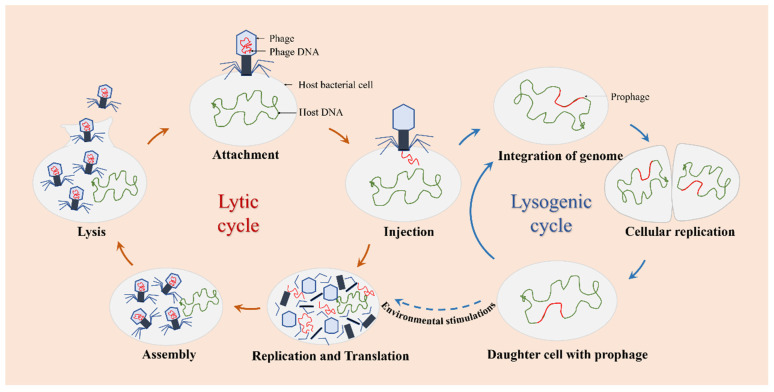
The life cycle of phages. Lytic phages only go through the lytic cycle, and lysogenic phages can go through the lytic or the lysogenic cycle.

The infection and replication of phages can be carried out through the lytic or lysogenic life cycle (Figure 2). Based on this cycle, there are two types of phages observed: lytic phages and lysogenic phages. Lytic phages, also known as virulent phages, typically undergo five stages of the lytic life cycle: attachment, injection, replication and translation, assembly, and lysis. Once infected with the host bacteria, lytic phages can replicate their genomes in a short time and achieve self-proliferation, leading to rapid cell destruction and lysis of the host cells [39]. Lysogenic phages, also known as temperate phages, refer to those phages that stably integrate themselves into the host genome as prophages during the lysogenic process [40,41]. Under environmental stimulations, prophages can exit the lysogenic state and become lytic.

As natural enemies of bacteria, phages can eradicate biofilms through several mechanisms and act on the target bacterial cells. One of the most crucial mechanisms is that phages can encode a variety of enzymes, such as depolymerases and lysins, to break down the defense barrier during infections of the host bacteria. For instance, Pires et al. have identified that there are 160 putative depolymerases in 143 phages, which can be divided into two main classes: hydrolases, including sialidase, levosidase, xylosidase, glucanase, rhamnosidase as well as peptidase; and lyases, including hyaluronidase, alginate lyase as well as pectin/pectin lyase [42]. These depolymerases are mostly found as free enzymes or tail-spike proteins of phages and can specifically recognize, bind, and digest EPSs of the host bacterial cells to disturb the biofilm structure, facilitating their penetration to the cells within the inner biofilm layers [43,44]. Lysins, also referred to as endolysins, are the general name of highly evolved peptidoglycan hydrolases produced towards the end of the lytic cycle of phage infection, which cause cell lysis and death by cleaving peptidoglycans in the bacterial cell wall and allowing the release of mature phage progenies from host cells [45].

### 3.2. Bacterial Biofilms Resist Phage Infections

**Figure 3:**
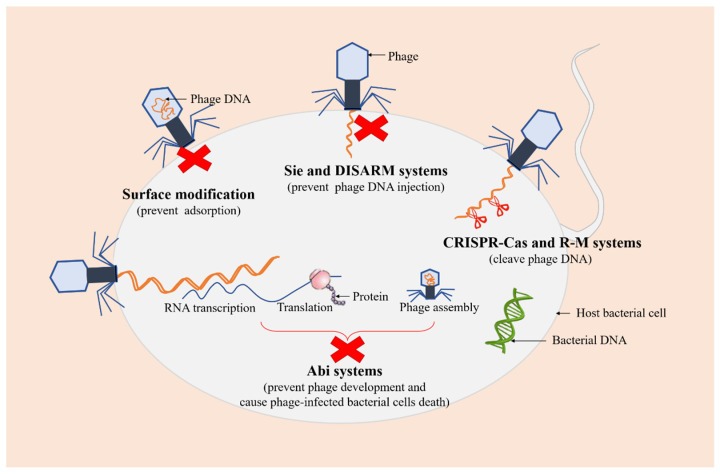
Several defense systems of bacteria against phages. The red crosses represent the arrest of the infection process. The green DNA molecule represents bacterial DNA. The orange DNA molecules represent phage DNA.

Recent studies have demonstrated that bacteria have evolved various defense mechanisms to cope with phage invasion, proliferation, and diffusion in order to survive phage infections, including surface modification, superinfection exclusion (Sie), restriction-modification (R-M) systems, and clustered regularly interspaced short palindromic repeats (CRISPR)-Cas, and abortive infection (Abi) systems [46,47] (Figure 3).

As a first-line bacterial defense, surface modification is considered to be one of the safest methods against phage predations, which can prevent the initial adsorption of phages to the cell. A previous study showed that glycosylated type IV pilin (T4P) with O-antigen units or polymers of D-arabinofuranose can block phage replication and protect *P. aeruginosa* from certain pilus-specific phages, suggesting that pilin glycosylation may represent a mechanism against phages using pili as a receptor [48]. Sie is a phenomenon in which a pre-existing viral infection prevents a secondary viral infection [49]. Phage-infected bacterial cells can quickly establish resistance against further infections with the same or closely related phages, thereby preventing the entry of phage DNA [50]. Additionally, a recent study has reported a novel defense system, a defense island system associated with restriction-modification (DISARM), to restrict incoming phage DNA [51].

R-M and CRISPR-Cas systems are two ubiquitous and extremely diverse defense mechanisms of bacteria against phages, which both recognize and cleave phage DNA at specific sites while protecting their own genomes [52]. R-M systems, mainly composed of restriction endonuclease and methyltransferase, are one of the tools commonly used by bacteria to preclude phage infection, which provide innate immunity against foreign DNA (such as DNA of phages) that lacks appropriate modification at specific recognition sites and protect the host genome from restriction endonuclease activity through the methylation of the same recognition sites [53,54]. It has been demonstrated that the ability of bacteria to defend against phages can be enhanced by increasing the concentration of restriction endonuclease in cells [55]. CRISPR-Cas systems are adaptive immune defense systems in bacteria, which protect prokaryotes from the invasion of phages and plasmids by recognizing and cleaving foreign nucleic acid sequences specified by CRISPR RNA spacer sequences, maintaining the integrity of their genomes [56,57].

Abi systems are considered to be the final barrier that cause bacterial cells to commit suicide after phage infections, also known as “altruistic cell suicide”, thereby reducing the spread of phages and protecting the overall bacterial population [58,59]. More specifically, Abi systems interfere with phage development and prevent its proliferation after phage adsorption and DNA injection into the host, resulting in the release of very few (if any) infectious virions, accompanied by the death of phage-infected bacterial cells [60].

## 4. Phage-Based Strategies for Preventing and Controlling Pathogenic Bacteria Biofilms

**Figure 4:**
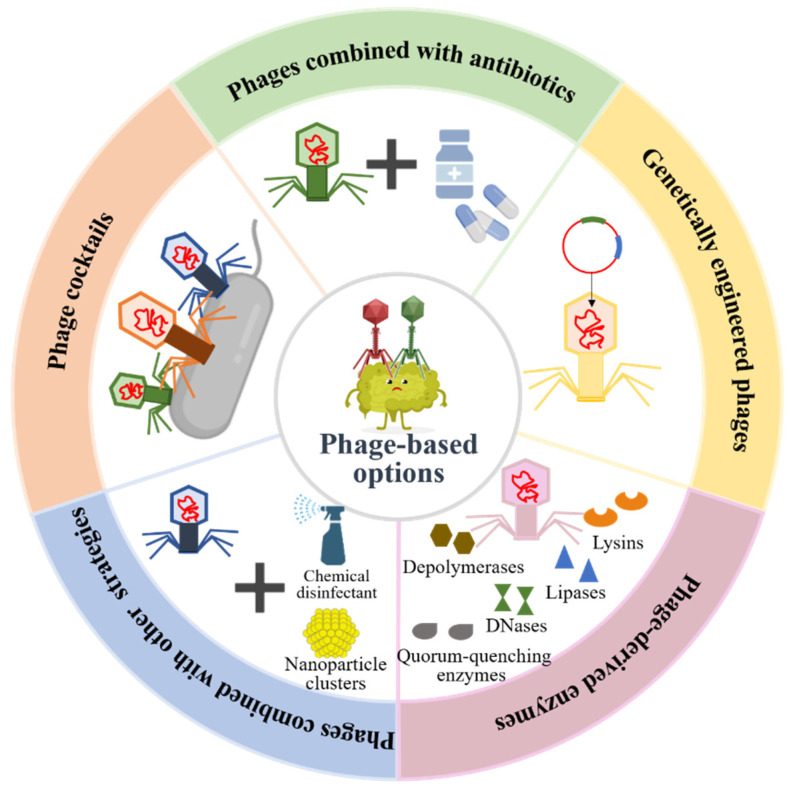
Phage-based therapeutic options in pathogenic bacterial biofilm preventions and destructions.

The increase in antibiotic-resistant bacterial infections is one of the major global public health challenges due to the abuse of antibiotics in modern medicine. According to the statistics, more than 700,000 people worldwide die of antibiotic resistance per year [61]. If effective measures are not taken, antibiotic resistance is predicted to cause 10 million deaths annually by 2050, which will exceed the number of cancer deaths and cause a total loss of USD 100 trillion to the world economy [62]. Therefore, there is an urgent need for the development of promising antimicrobial therapeutic alternatives to reduce the disease burden. Current studies have shown that phage therapy reveals great efficiency in the treatment of antibiotic-resistant bacterial infection caused by biofilms. Therapeutic options of phages and their derivatives in bacterial biofilm destructions are shown in Figure 4.

### 4.1. Phage Cocktail Therapy

Due to the modification of bacterial cell surface receptors, the generation of modified restriction enzymes that degrade phage DNA, and spontaneous mutation, the emergence of phage resistance may debilitate single-phage therapy [63]. Phage cocktails, a mixture of phages, can be used to effectively overcome the limitations of monophage therapy and improve treatment outcomes. Recent studies have demonstrated that phage cocktail therapy is more effective in preventing and eradicating pathogenic bacterial biofilms than individual phages. For instance, a study observed that a three-phage cocktail strongly inhibited biofilm formation and caused biofilm eradication of 2–3 *P. mirabilis* strains more compared to single phages without any inhibition to each other’s activity [64]. Moreover, it has been observed that the use of all four phages together in the form of a cocktail lysed 86.7% of the clinical isolates, compared to lysis in the range of 50–66% by individual phages, which indicated that phage cocktail therapy has a wider lytic spectrum than monophages [65]. In addition to broadening the host range and enhancing the lytic ability of phages, phage cocktails can also be used to significantly decrease the generation and mutation frequency of phage-resistant strains and maximize the efficiency of treatment of bacterial infection therapy [66,67]. Although phage cocktail therapy is more effective than monotherapy, they also carry a greater risk of unnecessary gene transfer and phage-to-phage interference [68]. Therefore, ideally, phages should be specifically tailored to their target pathogenic bacterial biofilms.

### 4.2. The Combination of Phage with Antibiotics

Phage therapy alone has proven to be active for clinical application. Recent studies have found that the practice of pairing phages with antibiotics could be one possible therapeutic approach to increase bacterial mortality and improve treatment efficacy. For example, the combination of phages with ciprofloxacin exhibited a tremendous synergistic effect, killing > 6 log CFUs/g of fibrin clots within 6 h and successfully treating 64% (*n* = 7/11) of rats with experimental endocarditis caused by *P. aeruginosa* [68]. It has been proposed that phages can cause a significant reduction in biofilm viability when used in conjunction with antimicrobial drugs compared to each treatment alone, displaying either synergy or facilitation [69]. Moreover, a previous study identified that the effect of treating biofilm infection can be significantly enhanced when biofilm is exposed to phages before antibiotics [70]. Similarly, a phage treatment preceding exposure to either vancomycin or cefazolin is more effective at eliminating *S. aureus* biofilm-associated cells, which may be related to the rapid replication of phages by treating biofilms with phages prior to antibiotics, resulting in high phage density and the destruction of the biofilm matrix, so that the subsequent addition of antibiotics can kill the bacteria more effectively [71]. Therefore, the order of therapeutic administration of phage combined with antibiotics to eliminate biofilms needs to be considered, and the precise mechanism between them is yet to be further elucidated.

### 4.3. Genetically Engineered Phages

In general, phage therapy uses phages from a variety of environments, while the application of natural phage therapy is limited due to their narrow host range and specificity [72]. Currently, it has become a research hotspot that phages can be modified by genetic engineering techniques to expand their host range, alter the host specificity, and increase biofilm degradation for much broader applications [73]. For instance, Li et al. obtained a recombinant T4-like phage named WGqlae by changing the receptor specificity determinant region of gene 37, conferring this engineered phage with the ability to lyse four additional hosts compared to its parental phages WG01 and QL01. In addition, phage WGqlae had a significant inhibitory effect on *E. coli* in the planktonic state and biofilm forms [74]. In a previous paper, Lu and Collins used engineered T7 phages that expressed dispersin B to simultaneously attack bacterial cells and facilitate the breakdown of the EPS of the *E. coli* biofilm, resulting in a significant reduction in the number of bacterial biofilm cells by 4.5 orders of magnitude and a substantial biofilm removal rate of about 99.997% [75]. Moreover, a study indicated that phage efficacy can be greatly enhanced by lysogenic to lytic conversion and single mutations, and its result demonstrated that several engineered phages, developed by genome engineering and forward genetics, can successfully treat disseminated drug-resistant *M. abscessus* infection [76]. In addition, Born and his colleagues obtained an engineered phage Y2::dpoL1-C by inserting the depolymerase dpoL1 gene into the genome of phage Y2, which greatly enhanced bacterial killing and had a positive effect on the dispersion of *E. amylovora* biofilm [77]. These studies suggested that genetically engineered phages may be promising candidates for future phage therapy applications against pathogenic biofilms.

### 4.4. Phage-Derived Enzymes

Natural and engineered phages are toolboxes offering an extensive arsenal of phage-borne enzymes, such as depolymerases, lysins, DNases, and lipases, which demonstrate an obvious inhibitory effect on the formation of pathogenic bacterial biofilm and a high lysis effect on existing biofilms [78]. Therefore, these phage-derived enzymes can be hailed as promising antibacterial agents in the prevention and eradication of infectious bacterial biofilms.

Some phages possess genes coding for extracellular polysaccharide depolymerases that can specifically degrade the polysaccharidic components of the EPS of biofilms and facilitate the access of phages to the deeper layers of the structure [44,79]. For instance, a recent study reported that a novel phage-derived depolymerase, Dpo10, can specifically bind and degrade lipopolysaccharide of *E. coli* O157 with moderate environmental stability and exhibit high activity to prevent biofilm formation on various abiotic surfaces [80]. Moreover, the O-specific polysaccharide lyase from the phage LKA1 tailspike which binds and cleaves B-band LPS was proven to efficiently reduce *P. aeruginosa* virulence in the in vivo *G. mellonella* infection model and effectively promote biofilm degradation without affecting the activity of ciprofloxacin and gentamicin [81]. In another study, Wu and his colleagues reported the potential application of a novel depolymerase Dep42 encoded by the phage SH-KP152226 in controlling infections caused by the K47 capsule of *K. pneumoniae*, and their results also suggested that there was a synergistic effect of the combined use of Dep42 and polymyxin against multidrug-resistant *K. pneumoniae* biofilms [82]. Additionally, a study demonstrated that recombinant depolymerase P510dep, a putative tail fiber protein with polysaccharide-degrading activity derived from *Przondovirus* phage P510, possessed high degradation activity against carbapenem-resistant *K. pneumoniae* biofilms [83]. Thus, depolymerases could be used as adjuvants in biofilm eradication.

Besides depolymerases, phage-derived lysins have been successfully used in the prevention and removal of bacterial biofilms, which could be applied in vivo as therapeutic agents. In a recent study, staphylococcus lysin P128 showed a significant reduction of up to 95.5% against the biofilm of methicillin-sensitive *S. aureus* and methicillin-resistant *S. aureus* isolated from the sinuses of chronic rhinosinusitis patients when applied in vitro at a concentration of ≥12.5 μg/mL [84]. Moreover, LysAB2 activity against multidrug-resistant *A. baumannii* was observed to increase by up to 100,000-fold through a two-step bacterial killing mechanism provided by its membrane-permeabilizing peptide CeA at the C-terminus, and the engineered LysAB2 also showed significant activity against *A. baumannii* and an outstanding capability to disrupt biofilm formation [85]. In another study, PM-477, an engineered phage-derived endolysin that is generated by domain shuffling, was reported to have the potential to completely kill *Gardnerella* bacteria and physically disrupt *Gardnerella*-dominated biofilms without damaging the remaining beneficial bacteria in patients with bacterial vaginosis [86]. In addition, phage-derived lysins could enhance intrinsic killing activity against multidrug-resistant strains when used in combination with other endolysins, antibiotics, or some compounds [45,87]. The synergistic effect of endolysin LysK and the poly-N-acetylglucosamine depolymerase DA7 used in combination can effectively degrade *S. aureus* biofilms, and it was confirmed in both static and dynamic models of infection [88].

In addition to the enzymes mentioned above, several other enzymes encoded by natural or engineered phages, such as DNases, quorum-quenching enzymes and lipases, are reported to have broad-range antibacterial effects and can be better exploited as biofilm-dispersing agents. Studies have shown that streptococcal-prophage-associated DNases, as virulence factors, play a major role in destroying extracellular traps produced by immune cells such as neutrophils, and may be able to eliminate biofilms formed by other competitive commensal bacteria or control the formation of their own biofilm [78,89]. In another paper, Pei et al. constructed an engineered T7 phage that expressed the quorum-quenching enzymes to effectively degrade acyl homoserine lactones (AHLs) from diverse bacteria and significantly inhibit the formation of mixed-species biofilms composed of *P. aeruginosa* and *E. coli* by interfering with quorum sensing [90]. Lipases are ubiquitous in nature, which are able to disperse biofilms by destroying the lipidic bounds involved in cell–cell or cell–surface interactions [78]. Indeed, lipase is a rare domain present in the structural components of phages. For example, Lipase_GDSL_3, a phage depolymerase domain with lipids hydrolysis activity, was found in eight *Cellulophaga* phages and one *Pseudomonas* phage [42].

### 4.5. The Combination of Phage with Other Strategies

Apart from the therapeutic options mentioned above, several other phage-based alternative strategies have also been reported in preventing and controlling bacterial biofilms. A current study on the potential synergistic effect of phage and chemical disinfection against the opportunistic pathogen *P. aeruginosa* has shown that phages can effectively combine with chemical disinfectants, such as sodium hypochlorite and benzalkonium chloride, to improve the removal of wet biofilms and bacterial spots on surfaces and prevent the regeneration of dry biofilms at the same time [91]. In addition, He et al. proposed a novel AIE-phage integrated strategy, in which phage PAP is equipped with photodynamic inactivation (PDI)-active AIEgens (luminogens with aggregation-induced emission property) to form a new type of antimicrobial bioconjugate, TVP-PAP, with a nearly 100% killing efficiency against multidrug-resistant *P. aeruginosa* [92].

With the development of green and biological nanotechnology, nanomaterials are becoming safer alternative antibiotic agents in eradicating pathogenic biofilms [93]. Recent studies have identified that phages can be immobilized onto nanocomposites by physical adsorption, based on electrostatic adhesion, or chemical binding [94]. It has been observed that the conjugation of polyvalent *Podoviridae* phages and magnetic colloidal nanoparticle clusters removed 98.3 ± 1.4% of the dual-species biofilm and 92.2 ± 3.1% of the multi-species biofilm coverage area after 6 h of treatment [95], whereas this binding approach can extend the application of phages in microbial control by enhancing their direct delivery to the relatively inaccessible inner layer of biofilms with the help of low-energy magnetic fields [96]. Furthermore, a study revealed that nano-TiO_2_ could promote phage gM13 attachment on the cell surface of *E. coli* TG1, which contributed to the infectious entry of phage gM13 [97].

## 5. Conclusions and Perspectives

Bacterial biofilms are shelters for bacteria, which significantly enhance the resistance of bacteria to antibiotics and the ability to escape from the host immune system, resulting in stubborn and recurrent infections. Due to the complexity of their compositions and antibiotic resistance mechanisms, biofilm communities are highly persistent and are difficult to be completely killed and removed by traditional antibiotic treatments. Increasing the dosage of antibiotics is not only ineffective for the eradication of biofilms but also leads to unexpected toxic and side effects.

Current studies and published patents have shown that phage-based therapies, including phage cocktails, phage and/or phage-derived products in combination with other antimicrobial agents, as well as engineered phages, are effective in controlling biofilms, targeting pathogens, and treating drug-resistant bacterial infections [98,99]. For instance, compositions containing an effective amount of phage-associated lysing enzymes and a carrier for delivering the lytic enzymes can be used to treat upper respiratory infections, skin infections, wounds, burns, vaginal infections, eye infections, intestinal disorders, and dental problems [100]. Another phage-derived lytic enzyme was disclosed to rapidly kill *S. pneumoniae* and other bacteria [101]. In addition, engineered homogeneous phage populations and chlorotoxin phages may be used for treating and/or imaging tumors, such as central nervous system tumors [102]. Regarding applications in food, phage therapy can be used against a variety of food pathogenic bacteria. For example, isolated phages can be used in various human and pet food products for the treatment or prevention of bacterial diseases caused by pathogenic *Enterobacteriaceae*, such as *E. coli* and *Salmonella* strains [103]. In fact, the FDA has recognized that some phage-based formulations as “generally considered safe” (GRAS) as food additives [79].

Though phages have great potential against pathogenic biofilms and bacterial infections caused by biofilms, there are still some unresolved issues. The first is the safety of phages and their derivatives. While no serious problems have been found in the current research, more basic and clinical studies are still needed in this area. In addition, the resistance of bacteria to phages and host immunity to phages also need to be further studied. Moreover, viruses are generally considered to be harmful to human health, and the use of phages in clinical therapy is not well recognized. In order to promote the public acceptance of phage-based therapies, it is of great necessity to gain government support and strengthen the publicity of phages.

## Data Availability

Not applicable.
